# *catena*-Poly[[tetra­kis(hexa­methyl­phos­pho­ramide-κ*O*)bis­(nitrato-κ^2^ *O*,*O*′)neodymium(III)] [silver(I)-di-μ_2_-sulfido-tungsten(VI)-di-μ_2_-sulfido]]

**DOI:** 10.1107/S1600536808001256

**Published:** 2008-02-15

**Authors:** Guodong Tang, Jinfang Zhang, Chi Zhang

**Affiliations:** a School of Chemical Engineering, Nanjing University of Science and Technology, 200 Xiaolingwei, Nanjing 210094, Jiangsu, People’s Republic of China; b Huaiyin Teachers’ College, Huaian 223001, Jiangsu, People’s Republic of China

## Abstract

In the title compound, {[Nd(NO_3_)_2_(C_6_H_18_N_3_OP)_4_][AgWS_4_]}_*n*_, the central Nd atom of the monovalent cation is coordinated by eight O atoms from two nitrate and four hexa­methyl­phospho­ramide ligands. The monovalent anion, {[WS_4_Ag]^−^}_*n*_, forms a polymeric chain in a distorted linear configuration with W—Ag—W and Ag—W—Ag angles of 163.81 (3) and 154.786 (12)°, respectively. Thirteen C and three N atoms are disordered equally over two positions. One C atom is disordered over two positions with site occupancy factors of 0.6 and 0.4.

## Related literature

For related structures, see: Lang *et al.* (1993 ▶); Huang *et al.* (1996 ▶, 1997 ▶); Zhang, Qian *et al.* (2007 ▶); Zhang, Cao *et al.* (2007 ▶). For a review of polymeric Mo(W)/S/Ag(Cu) clusters, see: Niu *et al.* (2004 ▶, and references therein). For third-order non-linear optical properties, see: Zhang, Song *et al.* (2007 ▶).
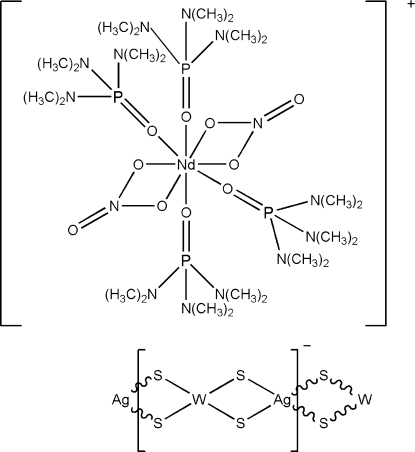


## Experimental

### 

#### Crystal data

[Nd(NO_3_)_2_(C_6_H_18_N_3_OP)_4_][AgWS_4_]
*M* *_r_* = 1405.07
Monoclinic, 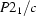

*a* = 15.8250 (19) Å
*b* = 29.873 (4) Å
*c* = 11.4345 (13) Å
β = 90.689 (3)°
*V* = 5405.2 (12) Å^3^
*Z* = 4
Mo *K*α radiation
μ = 3.76 mm^−1^
*T* = 153 (2) K
0.2 × 0.15 × 0.1 mm

#### Data collection

Rigaku Mercury diffractometer
Absorption correction: multi-scan (*SADABS*; Sheldrick, 1996 ▶) *T*_min_ = 0.132, *T*_max_ = 0.329 (expected range = 0.275–0.687)
46733 measured reflections
9877 independent reflections
9086 reflections with *I* > 2σ(*I*)
*R*_int_ = 0.045

#### Refinement

*R*[*F* ^2^ > 2σ(*F* ^2^)] = 0.050
*wR*(*F* ^2^) = 0.108
*S* = 1.14
9877 reflections
506 parameters
28 restraints
H-atom parameters constrained
Δρ_max_ = 1.42 e Å^−3^
Δρ_min_ = −1.36 e Å^−3^

### 

Data collection: *CrystalClear* (Rigaku Corporation, 2000 ▶); cell refinement: *CrystalClear*; data reduction: *CrystalStructure* (Rigaku/MSC, 2002 ▶); program(s) used to solve structure: *SHELXS97* (Sheldrick, 2008 ▶); program(s) used to refine structure: *SHELXL97* (Sheldrick, 2008 ▶); molecular graphics: *SHELXL97*; software used to prepare material for publication: *SHELXL97*.

## Supplementary Material

Crystal structure: contains datablocks I, global. DOI: 10.1107/S1600536808001256/pv2063sup1.cif

Structure factors: contains datablocks I. DOI: 10.1107/S1600536808001256/pv2063Isup2.hkl

Additional supplementary materials:  crystallographic information; 3D view; checkCIF report

## Figures and Tables

**Fig. 1.**
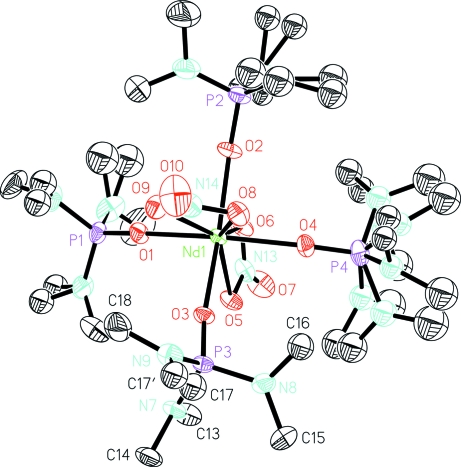
The molecular structure of the cation in the title compound, with atom labels and 30% probability displacement ellipsoids. All H atoms have been omitted for clarity.

**Fig. 2.**
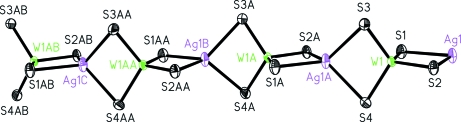
A portion of the anionic chain in the title compound, with atom labels and 30% probability displacement ellipsoids. (symmetry codes: i x, -y + 1/2, z - 1/2; ii x, y, z - 1; iii x, -y + 1/2, z - 1.5)

## supplementary crystallographic information

### Comment

One-dimensional Mo(W)/S/Ag anionic polymers have attracted much attention for their configurational isomerism (Niu *et al.*, 2004, and references therein) and potential applications, especially in third-order nonlinear optical (NLO) materials. (Zhang, Song *et al.*, 2007). Different solvent-coordinated rare-earth cations proved effective to obtain various configurations of anionic chains (Niu *et al.*, 2004, and references therein). The title compound, {[Nd(hmp)_4_(NO_3_)_2_][WS_4_Ag]}_n_ (hmp = hexamethylphosphoramide) with a wave-like anionic chain was prepared by following such route using Nd(III)-hmp complex as counterion.

In possession of two nitrate ligands, the cation in the title compound is univalent (Fig. 1), which leads to an anionic chain with a univalent repeat unit, unlike other solvent-coordinated rare-earth cations, in the literature (Niu *et al.*, 2004, and references therein), which are trivalent and induce trivalent repeat units. For example, [Nd(dmf)_8_]^3+^ induces an anionic chain with a trivalent repeat unit [W_4_S_16_Ag_5_]^3-^ (Huang *et al.*, 1996).

As illustrated in Fig. 2, the anionic chain in the title compound has a distorted linear configuration with W—Ag—W and Ag—W—Ag angles of 163.81 (3) and 154.786 (12) °, respectively, unlike those in {(*γ*-MePyH)[WS_4_Ag]}_n_ (Lang *et al.* 1993) and {[NH_3_C(CH_2_OH)_3_][WS_4_Ag](2DMF)}_n_ (Huang *et al.*, 1997), showing an ideal linear chain and a nearly linear chain, respectively. This observation suggests that cations with bigger bulk lead to more distorted anionic chains.

Similar angles for W—Ag—W and Ag—W—Ag are found in two distorted linear chains in {[Eu(hmp)_4_(NO_3_)_2_][WS_4_Ag]}_n_ (Zhang, Qian *et al.*, 2007) and {[Y(hmp)_4_(NO_3_)_2_][WS_4_Ag]}_n_ (Zhang, Cao *et al.*, 2007), implying that different rare earth cations with the same coordination environments will result in the same anionic structures.

### Experimental

Ag_2_S (0.25 g, 1 mmol) was added to a solution of (NH_4_)_2_WS_4_ (0.70 g, 2 mmol in 30 ml h mp) with thorough stirring for 10 h. The solution underwent an additional stir for one minute after Nd(NO_3_)_3_6H_2_O (0.44 g, 1 mmol) was added. After filtration the orange-red filtrate was carefully laid on the surface with 30 ml *i*-PrOH. Orange-red block crystals were obtained after ten days. Yield: 1.129 g in pure form, 40.2% (based on W).

### Refinement

H atoms were positioned geometrically and allowed in the refinements in a riding mode, with C—H bonds = 0.98 Å and *U*_iso_ = 1.5 times the *U*_eq_ of the parent C-atoms. Parts of dimethylamine groups from hmp ligands have large librations, resulting in some disordered C and N atoms. The occupancy factors for C19 and C19' were allowed to be 0.6 and 0.4, respectvely, and for the rest of the disordered atoms 0.5 and 0.5. The disordered non-hydrogen atoms (C1,C3,C5,C9,C10,C11, C12,C17,C19,C20,C21,C22,C23,C24,N10,N11,N12,) were allowed isotropic displacement parameters.

### Crystal data

**Table:**

[Nd(NO_3_)_2_(C_6_H_18_N_3_OP)_4_][AgWS_4_]	*F*_000_ = 2796.0
*M**_r_* = 1405.07	*D*_x_ = 1.727 Mg m^−^^3^
Monoclinic, *P*2_1_/*c*	Mo *K*α radiation λ = 0.71070 Å
Hall symbol: -P 2ybc	Cell parameters from 19261 reflections
*a* = 15.8250 (19) Å	θ = 3.0–25.4º
*b* = 29.873 (4) Å	µ = 3.76 mm^−^^1^
*c* = 11.4345 (13) Å	*T* = 153 (2) K
β = 90.689 (3)º	Block, orange–red
*V* = 5405.2 (12) Å^3^	0.2 × 0.15 × 0.1 mm
*Z* = 4	

### Data collection

**Table:**

Rigaku Mercury diffractometer	9877 independent reflections
Radiation source: fine-focus sealed tube	9086 reflections with *I* > 2σ(*I*)
Monochromator: graphite	*R*_int_ = 0.045
Detector resolution: 14.6306 pixels mm^-1^	θ_max_ = 25.4º
*T* = 153(2) K	θ_min_ = 3.0º
ω scans	*h* = −17→19
Absorption correction: multi-scan(SADABS; Sheldrick, 1996)	*k* = −35→35
*T*_min_ = 0.132, *T*_max_ = 0.329	*l* = −13→13
46733 measured reflections	

### Refinement

**Table:**

Refinement on *F*^2^	Secondary atom site location: difference Fourier map
Least-squares matrix: full	Hydrogen site location: inferred from neighbouring sites
*R*[*F*^2^ > 2σ(*F*^2^)] = 0.050	H-atom parameters constrained
*wR*(*F*^2^) = 0.108	*w* = 1/[σ^2^(*F*_o_^2^) + (0.0328*P*)^2^ + 29.5132*P*] where *P* = (*F*_o_^2^ + 2*F*_c_^2^)/3
*S* = 1.14	(Δ/σ)_max_ = 0.002
9877 reflections	Δρ_max_ = 1.42 e Å^−^^3^
506 parameters	Δρ_min_ = −1.36 e Å^−^^3^
28 restraints	Extinction correction: SHELXL97, Fc^*^=kFc[1+0.001xFc^2^λ^3^/sin(2θ)]^-1/4^
Primary atom site location: structure-invariant direct methods	Extinction coefficient: 0.00025 (5)

### Special details

**Table:**

Experimental. Analysis: calculated for C_24_H_72_AgN_14_NdO_10_P_4_S_4_W: C 20.52, H 5.16, N 13.96%; found: C 20.50, H 5.14, N 14.01%. IR: ν, cm^-1^, 478.5 m, 446.8 s (W-µ_2_-S).
Geometry. All e.s.d.'s (except the e.s.d. in the dihedral angle between two l.s. planes) are estimated using the full covariance matrix. The cell e.s.d.'s are taken into account individually in the estimation of e.s.d.'s in distances, angles and torsion angles; correlations between e.s.d.'s in cell parameters are only used when they are defined by crystal symmetry. An approximate (isotropic) treatment of cell e.s.d.'s is used for estimating e.s.d.'s involving l.s. planes.
Refinement. Refinement of *F*^2^ against ALL reflections. The weighted *R*-factor *wR* and goodness of fit *S* are based on *F*^2^, conventional *R*-factors *R* are based on *F*, with *F* set to zero for negative *F*^2^. The threshold expression of *F*^2^ > σ(*F*^2^) is used only for calculating *R*-factors(gt) *etc*. and is not relevant to the choice of reflections for refinement. *R*-factors based on *F*^2^ are statistically about twice as large as those based on *F*, and *R*- factors based on ALL data will be even larger.

### Fractional atomic coordinates and isotropic or equivalent isotropic displacement parameters (Å^2^)

**Table:**

	*x*	*y*	*z*	*U*_iso_*/*U*_eq_	Occ. (<1)
W1	0.713803 (18)	0.271347 (9)	−0.02311 (2)	0.03257 (10)	
Ag1	0.71456 (5)	0.23648 (2)	0.21805 (5)	0.05526 (19)	
S1	0.71465 (15)	0.19967 (7)	0.02245 (17)	0.0526 (5)	
S4	0.59999 (13)	0.28497 (8)	−0.12827 (18)	0.0544 (5)	
S3	0.82772 (13)	0.28620 (9)	−0.12453 (18)	0.0562 (6)	
S2	0.71231 (14)	0.31554 (6)	0.13071 (16)	0.0470 (5)	
Nd1	0.23703 (2)	0.083214 (11)	0.83521 (3)	0.03310 (11)	
P1	0.19278 (16)	−0.02955 (7)	0.69224 (19)	0.0531 (6)	
P2	0.46340 (12)	0.09454 (7)	0.7383 (2)	0.0509 (5)	
P3	0.01781 (11)	0.13540 (7)	0.82852 (17)	0.0392 (4)	
P4	0.29591 (17)	0.14957 (8)	1.1063 (2)	0.0604 (6)	
O1	0.2074 (3)	0.01663 (16)	0.7322 (5)	0.0508 (13)	
O4	0.2729 (3)	0.12885 (17)	0.9929 (5)	0.0504 (13)	
O2	0.3799 (3)	0.08064 (17)	0.7868 (5)	0.0521 (14)	
O3	0.0979 (3)	0.10903 (16)	0.8307 (5)	0.0430 (12)	
O5	0.1659 (3)	0.04041 (18)	1.0014 (5)	0.0517 (14)	
O6	0.2985 (3)	0.02619 (19)	0.9801 (6)	0.0581 (15)	
O7	0.2315 (5)	0.0018 (2)	1.1332 (7)	0.087 (2)	
O8	0.2510 (4)	0.15975 (17)	0.7396 (5)	0.0538 (14)	
O9	0.2232 (4)	0.10556 (19)	0.6219 (5)	0.0566 (14)	
O10	0.2227 (6)	0.1734 (3)	0.5559 (7)	0.108 (3)	
N1	0.2137 (6)	−0.0343 (3)	0.5527 (6)	0.074 (2)	
N2	0.2566 (7)	−0.0668 (3)	0.7514 (9)	0.090 (3)	
N3	0.1001 (6)	−0.0461 (3)	0.7286 (9)	0.101 (4)	
N4	0.5361 (4)	0.0702 (3)	0.8074 (9)	0.102 (4)	
N5	0.4625 (5)	0.0856 (4)	0.5961 (8)	0.090 (3)	
N6	0.4873 (5)	0.1474 (3)	0.7370 (8)	0.083 (3)	
N7	−0.0604 (4)	0.1016 (2)	0.8554 (6)	0.0487 (16)	
N8	0.0200 (4)	0.1751 (2)	0.9266 (7)	0.0588 (18)	
N9	0.0021 (4)	0.1589 (3)	0.7038 (6)	0.0602 (19)	
N13	0.2327 (5)	0.0221 (2)	1.0398 (7)	0.0567 (19)	
N14	0.2324 (5)	0.1468 (3)	0.6370 (7)	0.0596 (19)	
C2	0.2211 (10)	−0.0793 (4)	0.4976 (12)	0.126 (6)	
H2A	0.1672	−0.0873	0.4602	0.189*	
H2B	0.2355	−0.1015	0.5577	0.189*	
H2C	0.2655	−0.0786	0.4387	0.189*	
C4	0.2563 (13)	−0.0823 (6)	0.8638 (15)	0.160 (7)	
H4A	0.3027	−0.1037	0.8750	0.240*	
H4B	0.2024	−0.0972	0.8790	0.240*	
H4C	0.2637	−0.0572	0.9181	0.240*	
C6	0.0334 (7)	−0.0152 (4)	0.7584 (12)	0.099 (4)	
H6A	0.0540	0.0156	0.7507	0.149*	
H6B	0.0161	−0.0205	0.8393	0.149*	
H6C	−0.0150	−0.0198	0.7056	0.149*	
C7	0.5338 (10)	0.0962 (7)	0.5243 (13)	0.171 (9)	
H7A	0.5730	0.0708	0.5238	0.256*	
H7B	0.5628	0.1226	0.5560	0.256*	
H7C	0.5143	0.1024	0.4443	0.256*	
C8	0.4124 (8)	0.0485 (5)	0.5485 (12)	0.114 (5)	
H8A	0.4186	0.0474	0.4634	0.171*	
H8B	0.3528	0.0530	0.5676	0.171*	
H8C	0.4323	0.0203	0.5828	0.171*	
C13	−0.0526 (6)	0.0683 (3)	0.9472 (9)	0.072 (3)	
H13A	−0.1048	0.0507	0.9508	0.109*	
H13B	−0.0426	0.0832	1.0224	0.109*	
H13C	−0.0050	0.0484	0.9303	0.109*	
C14	−0.1476 (5)	0.1113 (4)	0.8189 (9)	0.070 (3)	
H14A	−0.1846	0.0868	0.8433	0.105*	
H14B	−0.1504	0.1145	0.7337	0.105*	
H14C	−0.1661	0.1393	0.8555	0.105*	
C15	−0.0477 (6)	0.1853 (4)	1.0071 (9)	0.077 (3)	
H15A	−0.0237	0.1921	1.0844	0.116*	
H15B	−0.0855	0.1594	1.0129	0.116*	
H15C	−0.0797	0.2112	0.9782	0.116*	
C16	0.0874 (7)	0.2085 (3)	0.9244 (11)	0.084 (3)	
H16A	0.0649	0.2368	0.8941	0.125*	
H16B	0.1329	0.1980	0.8738	0.125*	
H16C	0.1097	0.2130	1.0039	0.125*	
C18	0.0148 (7)	0.1332 (5)	0.5970 (9)	0.097 (4)	
H18A	0.0429	0.1049	0.6163	0.146*	
H18B	0.0502	0.1504	0.5434	0.146*	
H18C	−0.0400	0.1271	0.5595	0.146*	
N10	0.2744 (10)	0.2005 (5)	1.1022 (12)	0.062 (3)*	0.50
N10'	0.3460 (9)	0.1984 (5)	1.0899 (12)	0.062 (3)*	0.50
N11	0.4021 (11)	0.1396 (6)	1.1244 (16)	0.076 (3)*	0.50
N11'	0.3634 (11)	0.1297 (6)	1.1919 (15)	0.076 (3)*	0.50
N12	0.2128 (11)	0.1727 (6)	1.1823 (16)	0.083 (4)*	0.50
N12'	0.2418 (11)	0.1248 (6)	1.2046 (16)	0.083 (4)*	0.50
C1	0.1813 (16)	0.0006 (8)	0.477 (2)	0.085 (5)*	0.50
H1A	0.1972	−0.0057	0.3962	0.128*	0.50
H1B	0.2051	0.0294	0.5014	0.128*	0.50
H1C	0.1196	0.0017	0.4825	0.128*	0.50
C1'	0.2149 (16)	0.0014 (8)	0.471 (2)	0.085 (5)*	0.50
H1'A	0.2288	−0.0102	0.3939	0.128*	0.50
H1'B	0.2575	0.0234	0.4959	0.128*	0.50
H1'C	0.1591	0.0157	0.4683	0.128*	0.50
C3	0.3374 (16)	−0.0770 (15)	0.692 (3)	0.152 (10)*	0.50
H3A	0.3352	−0.0658	0.6120	0.227*	0.50
H3B	0.3463	−0.1095	0.6915	0.227*	0.50
H3C	0.3842	−0.0626	0.7350	0.227*	0.50
C3'	0.3492 (13)	−0.0640 (15)	0.743 (4)	0.152 (10)*	0.50
H3'1	0.3718	−0.0465	0.8087	0.227*	0.50
H3'2	0.3641	−0.0494	0.6692	0.227*	0.50
H3'3	0.3732	−0.0942	0.7449	0.227*	0.50
C5	0.0938 (15)	−0.0971 (6)	0.727 (2)	0.085 (5)*	0.50
H5A	0.0536	−0.1069	0.7868	0.128*	0.50
H5B	0.1495	−0.1101	0.7446	0.128*	0.50
H5C	0.0742	−0.1072	0.6501	0.128*	0.50
C5'	0.0559 (14)	−0.0855 (7)	0.690 (2)	0.085 (5)*	0.50
H5'A	0.0069	−0.0767	0.6420	0.128*	0.50
H5'B	0.0369	−0.1025	0.7580	0.128*	0.50
H5'C	0.0939	−0.1041	0.6433	0.128*	0.50
C9	0.6243 (10)	0.0721 (8)	0.771 (2)	0.081 (5)*	0.50
H9A	0.6548	0.0459	0.8009	0.121*	0.50
H9B	0.6507	0.0994	0.8011	0.121*	0.50
H9C	0.6266	0.0722	0.6849	0.121*	0.50
C9'	0.6235 (11)	0.0906 (8)	0.819 (2)	0.081 (5)*	0.50
H9'A	0.6517	0.0786	0.8895	0.121*	0.50
H9'B	0.6185	0.1232	0.8264	0.121*	0.50
H9'C	0.6567	0.0832	0.7503	0.121*	0.50
C10	0.5204 (14)	0.0235 (5)	0.8409 (18)	0.067 (4)*	0.50
H10D	0.5711	0.0056	0.8270	0.101*	0.50
H10E	0.4732	0.0116	0.7941	0.101*	0.50
H10F	0.5062	0.0222	0.9240	0.101*	0.50
C10'	0.5260 (14)	0.0337 (6)	0.8911 (16)	0.067 (4)*	0.50
H10G	0.5812	0.0259	0.9252	0.101*	0.50
H10H	0.5021	0.0075	0.8510	0.101*	0.50
H10I	0.4879	0.0432	0.9533	0.101*	0.50
C11	0.4938 (17)	0.1755 (8)	0.8378 (19)	0.097 (4)*	0.50
H11A	0.5079	0.2060	0.8135	0.145*	0.50
H11B	0.5382	0.1640	0.8903	0.145*	0.50
H11C	0.4397	0.1756	0.8787	0.145*	0.50
C11'	0.5275 (16)	0.1607 (9)	0.8511 (18)	0.097 (4)*	0.50
H11D	0.5413	0.1927	0.8493	0.145*	0.50
H11E	0.5794	0.1434	0.8637	0.145*	0.50
H11F	0.4882	0.1549	0.9149	0.145*	0.50
C12	0.4574 (17)	0.1859 (7)	0.677 (2)	0.097 (4)*	0.50
H12A	0.4896	0.2121	0.7036	0.145*	0.50
H12B	0.3974	0.1903	0.6943	0.145*	0.50
H12C	0.4645	0.1819	0.5930	0.145*	0.50
C12'	0.4557 (17)	0.1707 (8)	0.6275 (18)	0.097 (4)*	0.50
H12D	0.3978	0.1812	0.6394	0.145*	0.50
H12E	0.4566	0.1497	0.5617	0.145*	0.50
H12F	0.4924	0.1963	0.6105	0.145*	0.50
C17	−0.0382 (14)	0.2048 (6)	0.7073 (19)	0.070 (4)*	0.50
H17A	−0.0809	0.2236	0.6684	0.105*	0.50
H17B	0.0176	0.2188	0.6995	0.105*	0.50
H17C	−0.0517	0.2019	0.7903	0.105*	0.50
C17'	−0.0328 (14)	0.2003 (6)	0.6628 (19)	0.070 (4)*	0.50
H17D	−0.0137	0.2060	0.5829	0.105*	0.50
H17E	−0.0138	0.2247	0.7140	0.105*	0.50
H17F	−0.0946	0.1986	0.6634	0.105*	0.50
C19	0.2618 (17)	0.2274 (8)	1.207 (2)	0.122 (7)*	0.60
H19A	0.2956	0.2548	1.2019	0.184*	0.60
H19B	0.2796	0.2102	1.2760	0.184*	0.60
H19C	0.2019	0.2352	1.2132	0.184*	0.60
C19'	0.4180 (19)	0.2067 (14)	1.171 (3)	0.122 (7)*	0.40
H19D	0.4455	0.2350	1.1511	0.184*	0.40
H19E	0.4588	0.1822	1.1654	0.184*	0.40
H19F	0.3972	0.2084	1.2517	0.184*	0.40
C20	0.2952 (13)	0.2279 (8)	1.0033 (18)	0.065 (4)*	0.50
H20A	0.2886	0.2595	1.0238	0.098*	0.50
H20B	0.2574	0.2205	0.9375	0.098*	0.50
H20C	0.3538	0.2222	0.9810	0.098*	0.50
C20'	0.3282 (13)	0.2282 (7)	0.9940 (18)	0.065 (4)*	0.50
H20D	0.3497	0.2581	1.0126	0.098*	0.50
H20E	0.2670	0.2297	0.9804	0.098*	0.50
H20F	0.3557	0.2170	0.9234	0.098*	0.50
C21	0.4426 (17)	0.0973 (7)	1.097 (2)	0.093 (5)*	0.50
H21A	0.5031	0.0992	1.1159	0.139*	0.50
H21B	0.4350	0.0907	1.0137	0.139*	0.50
H21C	0.4170	0.0733	1.1434	0.139*	0.50
C21'	0.4250 (16)	0.0987 (8)	1.147 (2)	0.093 (5)*	0.50
H21D	0.4814	0.1122	1.1518	0.139*	0.50
H21E	0.4113	0.0918	1.0650	0.139*	0.50
H21F	0.4241	0.0711	1.1930	0.139*	0.50
C22	0.4572 (18)	0.1700 (10)	1.187 (3)	0.129 (7)*	0.50
H22A	0.5144	0.1575	1.1902	0.193*	0.50
H22B	0.4363	0.1745	1.2660	0.193*	0.50
H22C	0.4584	0.1988	1.1456	0.193*	0.50
C22'	0.330 (2)	0.1107 (10)	1.303 (2)	0.129 (7)*	0.50
H22D	0.3799	0.1096	1.3543	0.193*	0.50
H22E	0.2792	0.1148	1.3495	0.193*	0.50
H22F	0.3356	0.1358	1.2479	0.193*	0.50
C24'	0.259 (2)	0.1105 (12)	1.320 (2)	0.139 (9)*	0.50
H24C	0.2054	0.1037	1.3590	0.208*	0.50
H24E	0.2885	0.1343	1.3629	0.208*	0.50
H24F	0.2941	0.0836	1.3184	0.208*	0.50
C24	0.214 (2)	0.2198 (9)	1.222 (3)	0.139 (9)*	0.50
H24A	0.2631	0.2351	1.1901	0.208*	0.50
H24B	0.2167	0.2207	1.3078	0.208*	0.50
H24D	0.1621	0.2349	1.1951	0.208*	0.50
C23'	0.1513 (14)	0.1181 (11)	1.192 (3)	0.111 (6)*	0.50
H23A	0.1249	0.1201	1.2688	0.167*	0.50
H23D	0.1404	0.0885	1.1580	0.167*	0.50
H23B	0.1276	0.1412	1.1403	0.167*	0.50
C23	0.1510 (17)	0.1360 (9)	1.203 (3)	0.111 (6)*	0.50
H23C	0.1696	0.1088	1.1626	0.167*	0.50
H23G	0.0953	0.1449	1.1726	0.167*	0.50
H23E	0.1474	0.1300	1.2869	0.167*	0.50

### Atomic displacement parameters (Å^2^)

**Table:**

	*U*^11^	*U*^22^	*U*^33^	*U*^12^	*U*^13^	*U*^23^
W1	0.03803 (17)	0.03657 (17)	0.02301 (15)	0.00284 (12)	−0.00287 (11)	−0.00256 (11)
Ag1	0.0819 (5)	0.0585 (4)	0.0254 (3)	0.0011 (3)	−0.0014 (3)	0.0026 (3)
S1	0.0818 (15)	0.0361 (10)	0.0399 (10)	0.0074 (10)	−0.0006 (10)	−0.0064 (8)
S4	0.0440 (11)	0.0787 (15)	0.0403 (11)	0.0168 (10)	−0.0075 (9)	−0.0048 (10)
S3	0.0440 (11)	0.0866 (17)	0.0382 (11)	−0.0086 (11)	0.0012 (9)	−0.0017 (10)
S2	0.0686 (13)	0.0385 (10)	0.0340 (10)	−0.0017 (9)	−0.0014 (9)	−0.0090 (8)
Nd1	0.02623 (19)	0.02392 (19)	0.0491 (2)	0.00031 (14)	−0.00170 (16)	0.00114 (16)
P1	0.0735 (15)	0.0375 (11)	0.0485 (12)	−0.0155 (10)	0.0076 (11)	−0.0094 (9)
P2	0.0305 (10)	0.0497 (12)	0.0726 (15)	−0.0009 (9)	0.0057 (10)	0.0010 (11)
P3	0.0286 (9)	0.0433 (11)	0.0455 (11)	0.0068 (8)	−0.0020 (8)	0.0080 (9)
P4	0.0746 (16)	0.0554 (14)	0.0508 (13)	−0.0077 (12)	−0.0208 (12)	0.0031 (11)
O1	0.050 (3)	0.030 (3)	0.073 (4)	−0.002 (2)	−0.004 (3)	−0.008 (3)
O4	0.053 (3)	0.040 (3)	0.058 (3)	−0.002 (2)	−0.010 (3)	−0.007 (3)
O2	0.028 (3)	0.045 (3)	0.083 (4)	−0.002 (2)	0.008 (3)	0.010 (3)
O3	0.029 (2)	0.041 (3)	0.058 (3)	0.007 (2)	−0.003 (2)	0.000 (2)
O5	0.039 (3)	0.050 (3)	0.066 (4)	−0.003 (2)	−0.008 (3)	0.018 (3)
O6	0.040 (3)	0.047 (3)	0.087 (4)	0.000 (3)	−0.011 (3)	0.015 (3)
O7	0.087 (5)	0.086 (5)	0.089 (5)	−0.012 (4)	−0.018 (4)	0.047 (4)
O8	0.061 (4)	0.035 (3)	0.065 (4)	−0.009 (3)	−0.004 (3)	0.006 (3)
O9	0.070 (4)	0.047 (3)	0.053 (3)	−0.006 (3)	0.005 (3)	0.001 (3)
O10	0.169 (9)	0.073 (5)	0.079 (5)	−0.023 (5)	−0.023 (5)	0.039 (4)
N1	0.108 (7)	0.065 (5)	0.048 (4)	−0.025 (5)	0.007 (4)	−0.015 (4)
N2	0.128 (8)	0.039 (4)	0.102 (7)	0.008 (5)	0.031 (6)	0.007 (5)
N3	0.099 (7)	0.096 (7)	0.110 (7)	−0.063 (6)	0.042 (6)	−0.058 (6)
N4	0.033 (4)	0.140 (9)	0.133 (8)	0.010 (5)	0.007 (4)	0.089 (7)
N5	0.062 (5)	0.139 (9)	0.070 (6)	−0.022 (6)	0.013 (4)	0.007 (6)
N6	0.062 (5)	0.073 (6)	0.115 (8)	−0.007 (4)	0.014 (5)	0.022 (5)
N7	0.027 (3)	0.063 (4)	0.056 (4)	0.001 (3)	−0.003 (3)	0.011 (3)
N8	0.048 (4)	0.056 (4)	0.073 (5)	0.007 (3)	0.009 (4)	−0.010 (4)
N9	0.052 (4)	0.074 (5)	0.054 (4)	0.004 (4)	−0.006 (3)	0.020 (4)
N13	0.060 (5)	0.044 (4)	0.066 (5)	−0.010 (3)	−0.021 (4)	0.014 (4)
N14	0.064 (5)	0.051 (5)	0.064 (5)	−0.008 (4)	0.003 (4)	0.016 (4)
C2	0.167 (14)	0.102 (10)	0.112 (11)	−0.047 (9)	0.064 (10)	−0.058 (8)
C4	0.22 (2)	0.128 (14)	0.132 (14)	0.072 (14)	−0.005 (14)	0.029 (11)
C6	0.061 (7)	0.112 (10)	0.125 (11)	−0.011 (7)	−0.019 (7)	0.019 (8)
C7	0.127 (13)	0.29 (3)	0.100 (12)	−0.083 (15)	0.032 (10)	−0.004 (13)
C8	0.082 (8)	0.151 (14)	0.109 (10)	−0.016 (9)	0.004 (7)	−0.039 (9)
C13	0.055 (5)	0.077 (7)	0.085 (7)	−0.001 (5)	−0.001 (5)	0.031 (6)
C14	0.032 (4)	0.100 (8)	0.078 (7)	−0.004 (5)	−0.002 (4)	0.006 (6)
C15	0.075 (7)	0.080 (7)	0.077 (7)	0.015 (6)	0.017 (5)	−0.017 (6)
C16	0.074 (7)	0.061 (6)	0.116 (9)	−0.002 (5)	0.007 (6)	−0.027 (6)
C18	0.076 (7)	0.149 (12)	0.065 (7)	−0.005 (8)	−0.016 (6)	0.002 (7)

### Geometric parameters (Å, °)

**Table:**

W1—S4	2.192 (2)	C16—H16C	0.9800
W1—S2	2.1997 (18)	C18—H18A	0.9800
W1—S3	2.200 (2)	C18—H18B	0.9800
W1—S1	2.204 (2)	C18—H18C	0.9800
W1—Ag1	2.9477 (7)	N10—C20	1.436 (16)
W1—Ag1^i^	2.9690 (7)	N10—C19	1.457 (17)
Ag1—S1	2.492 (2)	N10'—C20'	1.437 (16)
Ag1—S2	2.565 (2)	N10'—C19'	1.484 (18)
Ag1—S3^ii^	2.613 (2)	N11—C22	1.441 (17)
Ag1—S4^ii^	2.620 (2)	N11—C21	1.453 (17)
Ag1—W1^ii^	2.9690 (7)	N11'—C21'	1.444 (17)
S4—Ag1^i^	2.620 (2)	N12—C24	1.481 (18)
S3—Ag1^i^	2.613 (2)	N12—C23	1.490 (18)
Nd1—O4	2.325 (5)	N12'—C24'	1.410 (18)
Nd1—O3	2.333 (4)	N12'—C23'	1.451 (18)
Nd1—O2	2.335 (5)	C1—H1A	0.9800
Nd1—O1	2.356 (5)	C1—H1B	0.9800
Nd1—O9	2.535 (6)	C1—H1C	0.9800
Nd1—O8	2.545 (5)	C1'—H1'A	0.9800
Nd1—O6	2.560 (5)	C1'—H1'B	0.9800
Nd1—O5	2.562 (5)	C1'—H1'C	0.9800
Nd1—N14	2.957 (7)	C3—H3A	0.9800
Nd1—N13	2.968 (7)	C3—H3B	0.9800
P1—O1	1.471 (5)	C3—H3C	0.9800
P1—N3	1.608 (9)	C3'—H3'1	0.9800
P1—N1	1.639 (8)	C3'—H3'2	0.9800
P1—N2	1.642 (10)	C3'—H3'3	0.9800
P2—O2	1.498 (5)	C5—H5A	0.9800
P2—N4	1.568 (8)	C5—H5B	0.9800
P2—N6	1.623 (9)	C5—H5C	0.9800
P2—N5	1.647 (9)	C5'—H5'A	0.9800
P3—O3	1.492 (5)	C5'—H5'B	0.9800
P3—N9	1.607 (7)	C5'—H5'C	0.9800
P3—N7	1.630 (7)	C9—H9A	0.9800
P3—N8	1.632 (7)	C9—H9B	0.9800
P4—O4	1.479 (6)	C9—H9C	0.9800
P4—N11'	1.557 (17)	C9'—H9'A	0.9800
P4—N10	1.561 (15)	C9'—H9'B	0.9800
P4—N12'	1.602 (18)	C9'—H9'C	0.9800
P4—N10'	1.673 (15)	C10—H10D	0.9800
P4—N11	1.716 (17)	C10—H10E	0.9800
P4—N12	1.729 (18)	C10—H10F	0.9800
O5—N13	1.263 (8)	C10'—H10G	0.9800
O6—N13	1.258 (9)	C10'—H10H	0.9800
O7—N13	1.230 (9)	C10'—H10I	0.9800
O8—N14	1.266 (9)	C11—H11A	0.9800
O9—N14	1.252 (9)	C11—H11B	0.9800
O10—N14	1.230 (9)	C11—H11C	0.9800
N1—C1'	1.415 (16)	C11'—H11D	0.9800
N1—C1	1.444 (16)	C11'—H11E	0.9800
N1—C2	1.489 (13)	C11'—H11F	0.9800
N2—C4	1.367 (17)	C12—H12A	0.9800
N2—C3'	1.472 (18)	C12—H12B	0.9800
N2—C3	1.485 (18)	C12—H12C	0.9800
N3—C5'	1.435 (16)	C12'—H12D	0.9800
N3—C6	1.446 (14)	C12'—H12E	0.9800
N3—C5	1.528 (16)	C12'—H12F	0.9800
N4—C10'	1.461 (15)	C17—H17A	0.9800
N4—C9	1.464 (15)	C17—H17B	0.9800
N4—C10	1.468 (15)	C17—H17C	0.9800
N4—C9'	1.515 (15)	C17'—H17D	0.9800
N5—C7	1.438 (15)	C17'—H17E	0.9800
N5—C8	1.465 (15)	C17'—H17F	0.9800
N6—C12	1.416 (16)	C19—H19A	0.9800
N6—C11	1.428 (16)	C19—H19B	0.9800
N6—C11'	1.498 (16)	C19—H19C	0.9800
N6—C12'	1.512 (16)	C19'—H19D	0.9800
N7—C13	1.450 (11)	C19'—H19E	0.9800
N7—C14	1.466 (10)	C19'—H19F	0.9800
N8—C15	1.453 (11)	C20—H20A	0.9800
N8—C16	1.461 (12)	C20—H20B	0.9800
N9—C17'	1.431 (15)	C20—H20C	0.9800
N9—C18	1.459 (13)	C20'—H20D	0.9800
C2—H2A	0.9800	C20'—H20E	0.9800
C2—H2B	0.9800	C20'—H20F	0.9800
C2—H2C	0.9800	C21—H21A	0.9800
C4—H4A	0.9800	C21—H21B	0.9800
C4—H4B	0.9800	C21—H21C	0.9800
C4—H4C	0.9800	C21'—H21D	0.9800
C6—H6A	0.9800	C21'—H21E	0.9800
C6—H6B	0.9800	C21'—H21F	0.9800
C6—H6C	0.9800	C22—H22A	0.9800
C7—H7A	0.9800	C22—H22B	0.9800
C7—H7B	0.9800	C22—H22C	0.9800
C7—H7C	0.9800	C22'—H22D	0.9800
C8—H8A	0.9800	C22'—H22E	0.9800
C8—H8B	0.9800	C22'—H22F	0.9800
C8—H8C	0.9800	C24'—H24C	0.9800
C13—H13A	0.9800	C24'—H24E	0.9800
C13—H13B	0.9800	C24'—H24F	0.9800
C13—H13C	0.9800	C24—H24A	0.9800
C14—H14A	0.9800	C24—H24B	0.9800
C14—H14B	0.9800	C24—H24D	0.9800
C14—H14C	0.9800	C23'—H23A	0.9800
C15—H15A	0.9800	C23'—H23D	0.9800
C15—H15B	0.9800	C23'—H23B	0.9800
C15—H15C	0.9800	C23—H23C	0.9800
C16—H16A	0.9800	C23—H23G	0.9800
C16—H16B	0.9800	C23—H23E	0.9800
			
S4—W1—S2	108.09 (8)	N7—C13—H13A	109.5
S4—W1—S3	110.27 (8)	N7—C13—H13B	109.5
S2—W1—S3	108.49 (8)	H13A—C13—H13B	109.5
S4—W1—S1	108.23 (9)	N7—C13—H13C	109.5
S2—W1—S1	113.22 (7)	H13A—C13—H13C	109.5
S3—W1—S1	108.53 (9)	H13B—C13—H13C	109.5
S4—W1—Ag1	124.95 (6)	N7—C14—H14A	109.5
S2—W1—Ag1	57.59 (5)	N7—C14—H14B	109.5
S3—W1—Ag1	124.76 (6)	H14A—C14—H14B	109.5
S1—W1—Ag1	55.63 (5)	N7—C14—H14C	109.5
S4—W1—Ag1^i^	58.73 (6)	H14A—C14—H14C	109.5
S2—W1—Ag1^i^	147.62 (6)	H14B—C14—H14C	109.5
S3—W1—Ag1^i^	58.49 (6)	N8—C15—H15A	109.5
S1—W1—Ag1^i^	99.16 (5)	N8—C15—H15B	109.5
Ag1—W1—Ag1^i^	154.786 (12)	H15A—C15—H15B	109.5
S1—Ag1—S2	93.26 (7)	N8—C15—H15C	109.5
S1—Ag1—S3^ii^	119.74 (8)	H15A—C15—H15C	109.5
S2—Ag1—S3^ii^	120.87 (8)	H15B—C15—H15C	109.5
S1—Ag1—S4^ii^	120.13 (8)	N8—C16—H16A	109.5
S2—Ag1—S4^ii^	118.69 (8)	N8—C16—H16B	109.5
S3^ii^—Ag1—S4^ii^	87.06 (7)	H16A—C16—H16B	109.5
S1—Ag1—W1	46.87 (5)	N8—C16—H16C	109.5
S2—Ag1—W1	46.40 (4)	H16A—C16—H16C	109.5
S3^ii^—Ag1—W1	136.97 (5)	H16B—C16—H16C	109.5
S4^ii^—Ag1—W1	135.97 (5)	N9—C18—H18A	109.5
S1—Ag1—W1^ii^	149.31 (5)	N9—C18—H18B	109.5
S2—Ag1—W1^ii^	117.42 (5)	H18A—C18—H18B	109.5
S3^ii^—Ag1—W1^ii^	45.88 (5)	N9—C18—H18C	109.5
S4^ii^—Ag1—W1^ii^	45.65 (4)	H18A—C18—H18C	109.5
W1—Ag1—W1^ii^	163.81 (3)	H18B—C18—H18C	109.5
W1—S1—Ag1	77.50 (6)	C20—N10—C19	111.7 (18)
W1—S4—Ag1^i^	75.63 (6)	C20—N10—P4	121.7 (14)
W1—S3—Ag1^i^	75.63 (6)	C19—N10—P4	123.0 (15)
W1—S2—Ag1	76.02 (6)	C20'—N10'—C19'	121 (2)
O4—Nd1—O3	92.58 (18)	C20'—N10'—P4	122.4 (13)
O4—Nd1—O2	88.6 (2)	C19'—N10'—P4	115.9 (18)
O3—Nd1—O2	157.18 (18)	C22—N11—C21	113 (2)
O4—Nd1—O1	158.12 (19)	C22—N11—P4	122.4 (17)
O3—Nd1—O1	94.92 (18)	C21—N11—P4	123.9 (16)
O2—Nd1—O1	92.36 (19)	C21'—N11'—P4	118.7 (16)
O4—Nd1—O9	127.51 (19)	C24—N12—C23	131 (2)
O3—Nd1—O9	79.69 (19)	C24—N12—P4	122.0 (18)
O2—Nd1—O9	81.6 (2)	C23—N12—P4	106.9 (17)
O1—Nd1—O9	74.15 (19)	C24'—N12'—C23'	103 (2)
O4—Nd1—O8	77.58 (19)	C24'—N12'—P4	134.4 (19)
O3—Nd1—O8	77.30 (18)	C23'—N12'—P4	121.9 (19)
O2—Nd1—O8	80.71 (18)	N1—C1—H1A	109.5
O1—Nd1—O8	124.14 (19)	N1—C1—H1B	109.5
O9—Nd1—O8	49.99 (18)	H1A—C1—H1B	109.5
O4—Nd1—O6	78.53 (19)	N1—C1—H1C	109.5
O3—Nd1—O6	125.83 (19)	H1A—C1—H1C	109.5
O2—Nd1—O6	76.72 (19)	H1B—C1—H1C	109.5
O1—Nd1—O6	80.42 (19)	N1—C1'—H1'A	109.5
O9—Nd1—O6	145.6 (2)	N1—C1'—H1'B	109.5
O8—Nd1—O6	147.32 (18)	H1'A—C1'—H1'B	109.5
O4—Nd1—O5	79.80 (19)	N1—C1'—H1'C	109.5
O3—Nd1—O5	76.02 (17)	H1'A—C1'—H1'C	109.5
O2—Nd1—O5	126.49 (18)	H1'B—C1'—H1'C	109.5
O1—Nd1—O5	82.12 (19)	N2—C3—H3A	109.5
O9—Nd1—O5	144.21 (18)	N2—C3—H3B	109.5
O8—Nd1—O5	143.91 (19)	H3A—C3—H3B	109.5
O6—Nd1—O5	49.82 (17)	N2—C3—H3C	109.5
O4—Nd1—N14	102.8 (2)	H3A—C3—H3C	109.5
O3—Nd1—N14	75.88 (19)	H3B—C3—H3C	109.5
O2—Nd1—N14	81.6 (2)	N2—C3'—H3'1	109.5
O1—Nd1—N14	99.0 (2)	N2—C3'—H3'2	109.5
O9—Nd1—N14	24.87 (19)	H3'1—C3'—H3'2	109.5
O8—Nd1—N14	25.19 (19)	N2—C3'—H3'3	109.5
O6—Nd1—N14	158.3 (2)	H3'1—C3'—H3'3	109.5
O5—Nd1—N14	151.87 (19)	H3'2—C3'—H3'3	109.5
O4—Nd1—N13	75.94 (19)	N3—C5—H5A	109.5
O3—Nd1—N13	100.9 (2)	N3—C5—H5B	109.5
O2—Nd1—N13	101.4 (2)	H5A—C5—H5B	109.5
O1—Nd1—N13	82.5 (2)	N3—C5—H5C	109.5
O9—Nd1—N13	156.55 (19)	H5A—C5—H5C	109.5
O8—Nd1—N13	153.4 (2)	H5B—C5—H5C	109.5
O6—Nd1—N13	24.93 (18)	N3—C5'—H5'A	109.5
O5—Nd1—N13	25.05 (18)	N3—C5'—H5'B	109.5
N14—Nd1—N13	176.6 (2)	H5'A—C5'—H5'B	109.5
O1—P1—N3	110.4 (4)	N3—C5'—H5'C	109.5
O1—P1—N1	110.5 (4)	H5'A—C5'—H5'C	109.5
N3—P1—N1	114.9 (5)	H5'B—C5'—H5'C	109.5
O1—P1—N2	114.4 (4)	N4—C9—H9A	109.5
N3—P1—N2	104.1 (6)	N4—C9—H9B	109.5
N1—P1—N2	102.2 (5)	H9A—C9—H9B	109.5
O2—P2—N4	109.3 (4)	N4—C9—H9C	109.5
O2—P2—N6	118.6 (4)	H9A—C9—H9C	109.5
N4—P2—N6	106.6 (5)	H9B—C9—H9C	109.5
O2—P2—N5	108.9 (4)	N4—C9'—H9'A	109.5
N4—P2—N5	114.8 (6)	N4—C9'—H9'B	109.5
N6—P2—N5	98.5 (5)	H9'A—C9'—H9'B	109.5
O3—P3—N9	111.6 (3)	N4—C9'—H9'C	109.5
O3—P3—N7	108.4 (3)	H9'A—C9'—H9'C	109.5
N9—P3—N7	109.2 (4)	H9'B—C9'—H9'C	109.5
O3—P3—N8	111.1 (3)	N4—C10—H10D	109.5
N9—P3—N8	107.1 (4)	N4—C10—H10E	109.5
N7—P3—N8	109.3 (4)	H10D—C10—H10E	109.5
O4—P4—N11'	123.5 (7)	N4—C10—H10F	109.5
O4—P4—N10	109.3 (6)	H10D—C10—H10F	109.5
N11'—P4—N10	122.5 (8)	H10E—C10—H10F	109.5
O4—P4—N12'	107.1 (7)	N4—C10'—H10G	109.5
N11'—P4—N12'	75.6 (9)	N4—C10'—H10H	109.5
N10—P4—N12'	110.7 (9)	H10G—C10'—H10H	109.5
O4—P4—N10'	112.3 (6)	N4—C10'—H10I	109.5
N11'—P4—N10'	94.6 (8)	H10G—C10'—H10I	109.5
N12'—P4—N10'	137.7 (8)	H10H—C10'—H10I	109.5
O4—P4—N11	105.3 (6)	N6—C11—H11A	109.5
N10—P4—N11	112.7 (8)	N6—C11—H11B	109.5
N12'—P4—N11	111.5 (9)	H11A—C11—H11B	109.5
N10'—P4—N11	72.6 (8)	N6—C11—H11C	109.5
O4—P4—N12	115.3 (6)	H11A—C11—H11C	109.5
N11'—P4—N12	110.9 (9)	H11B—C11—H11C	109.5
N10—P4—N12	57.2 (8)	N6—C11'—H11D	109.5
N12'—P4—N12	54.2 (8)	N6—C11'—H11E	109.5
N10'—P4—N12	94.1 (8)	H11D—C11'—H11E	109.5
N11—P4—N12	139.3 (8)	N6—C11'—H11F	109.5
P1—O1—Nd1	167.7 (4)	H11D—C11'—H11F	109.5
P4—O4—Nd1	168.7 (4)	H11E—C11'—H11F	109.5
P2—O2—Nd1	160.0 (3)	N6—C12—H12A	109.5
P3—O3—Nd1	167.4 (3)	N6—C12—H12B	109.5
N13—O5—Nd1	95.8 (4)	H12A—C12—H12B	109.5
N13—O6—Nd1	96.0 (4)	N6—C12—H12C	109.5
N14—O8—Nd1	95.9 (4)	H12A—C12—H12C	109.5
N14—O9—Nd1	96.8 (5)	H12B—C12—H12C	109.5
C1'—N1—C2	113.6 (14)	N6—C12'—H12D	109.5
C1—N1—C2	115.3 (14)	N6—C12'—H12E	109.5
C1'—N1—P1	125.3 (13)	H12D—C12'—H12E	109.5
C1—N1—P1	116.5 (13)	N6—C12'—H12F	109.5
C2—N1—P1	120.5 (8)	H12D—C12'—H12F	109.5
C4—N2—C3'	96 (2)	H12E—C12'—H12F	109.5
C4—N2—C3	112 (2)	H17A—C17—H17B	109.5
C4—N2—P1	127.5 (10)	H17A—C17—H17C	109.5
C3'—N2—P1	122.8 (19)	H17B—C17—H17C	109.5
C3—N2—P1	118.8 (19)	N9—C17'—H17D	109.5
C5'—N3—C6	104.0 (13)	N9—C17'—H17E	109.5
C6—N3—C5	126.3 (12)	H17D—C17'—H17E	109.5
C5'—N3—P1	127.9 (12)	N9—C17'—H17F	109.5
C6—N3—P1	122.3 (8)	H17D—C17'—H17F	109.5
C5—N3—P1	111.4 (11)	H17E—C17'—H17F	109.5
C10'—N4—C9	109.3 (14)	N10—C19—H19A	109.5
C9—N4—C10	106.1 (14)	N10—C19—H19B	109.5
C10'—N4—C9'	110.4 (14)	H19A—C19—H19B	109.5
C10—N4—C9'	121.1 (14)	N10—C19—H19C	109.5
C10'—N4—P2	126.2 (10)	H19A—C19—H19C	109.5
C9—N4—P2	122.3 (11)	H19B—C19—H19C	109.5
C10—N4—P2	116.5 (10)	N10'—C19'—H19D	109.5
C9'—N4—P2	121.5 (11)	N10'—C19'—H19E	109.5
C7—N5—C8	112.3 (11)	H19D—C19'—H19E	109.5
C7—N5—P2	122.0 (9)	N10'—C19'—H19F	109.5
C8—N5—P2	119.1 (8)	H19D—C19'—H19F	109.5
C12—N6—C11	86.0 (16)	H19E—C19'—H19F	109.5
C12—N6—C11'	109.8 (16)	N10—C20—H20A	109.5
C11—N6—C12'	114.6 (16)	N10—C20—H20B	109.5
C11'—N6—C12'	136.9 (17)	H20A—C20—H20B	109.5
C12—N6—P2	135.9 (13)	N10—C20—H20C	109.5
C11—N6—P2	125.4 (13)	H20A—C20—H20C	109.5
C11'—N6—P2	110.4 (12)	H20B—C20—H20C	109.5
C12'—N6—P2	112.4 (12)	N10'—C20'—H20D	109.5
C13—N7—C14	114.5 (7)	N10'—C20'—H20E	109.5
C13—N7—P3	120.2 (5)	H20D—C20'—H20E	109.5
C14—N7—P3	122.5 (6)	N10'—C20'—H20F	109.5
C15—N8—C16	114.3 (8)	H20D—C20'—H20F	109.5
C15—N8—P3	125.3 (7)	H20E—C20'—H20F	109.5
C16—N8—P3	119.6 (6)	N11—C21—H21A	109.5
C17'—N9—C18	103.7 (11)	N11—C21—H21B	109.5
C17'—N9—P3	136.4 (11)	H21A—C21—H21B	109.5
C18—N9—P3	119.4 (7)	N11—C21—H21C	109.5
O7—N13—O6	122.7 (7)	H21A—C21—H21C	109.5
O7—N13—O5	119.6 (8)	H21B—C21—H21C	109.5
O6—N13—O5	117.6 (7)	N11'—C21'—H21D	109.5
O7—N13—Nd1	171.7 (6)	N11'—C21'—H21E	109.5
O6—N13—Nd1	59.0 (4)	H21D—C21'—H21E	109.5
O5—N13—Nd1	59.2 (4)	N11'—C21'—H21F	109.5
O10—N14—O9	121.2 (8)	H21D—C21'—H21F	109.5
O10—N14—O8	121.8 (8)	H21E—C21'—H21F	109.5
O9—N14—O8	117.0 (7)	N11—C22—H22A	109.5
O10—N14—Nd1	174.2 (7)	N11—C22—H22B	109.5
O9—N14—Nd1	58.4 (4)	H22A—C22—H22B	109.5
O8—N14—Nd1	58.9 (4)	N11—C22—H22C	109.5
N1—C2—H2A	109.5	H22A—C22—H22C	109.5
N1—C2—H2B	109.5	H22B—C22—H22C	109.5
H2A—C2—H2B	109.5	H22D—C22'—H22E	109.5
N1—C2—H2C	109.5	H22D—C22'—H22F	109.5
H2A—C2—H2C	109.5	H22E—C22'—H22F	109.5
H2B—C2—H2C	109.5	N12'—C24'—H24C	109.5
N2—C4—H4A	109.5	N12'—C24'—H24E	109.5
N2—C4—H4B	109.5	H24C—C24'—H24E	109.5
H4A—C4—H4B	109.5	N12'—C24'—H24F	109.5
N2—C4—H4C	109.5	H24C—C24'—H24F	109.5
H4A—C4—H4C	109.5	H24E—C24'—H24F	109.5
H4B—C4—H4C	109.5	N12—C24—H24A	109.5
N3—C6—H6A	109.5	N12—C24—H24B	109.5
N3—C6—H6B	109.5	H24A—C24—H24B	109.5
H6A—C6—H6B	109.5	N12—C24—H24D	109.5
N3—C6—H6C	109.5	H24A—C24—H24D	109.5
H6A—C6—H6C	109.5	H24B—C24—H24D	109.5
H6B—C6—H6C	109.5	N12'—C23'—H23A	109.5
N5—C7—H7A	109.5	N12'—C23'—H23D	109.5
N5—C7—H7B	109.5	H23A—C23'—H23D	109.5
H7A—C7—H7B	109.5	N12'—C23'—H23B	109.5
N5—C7—H7C	109.5	H23A—C23'—H23B	109.5
H7A—C7—H7C	109.5	H23D—C23'—H23B	109.5
H7B—C7—H7C	109.5	N12—C23—H23C	109.5
N5—C8—H8A	109.5	N12—C23—H23G	109.5
N5—C8—H8B	109.5	H23C—C23—H23G	109.5
H8A—C8—H8B	109.5	N12—C23—H23E	109.5
N5—C8—H8C	109.5	H23C—C23—H23E	109.5
H8A—C8—H8C	109.5	H23G—C23—H23E	109.5
H8B—C8—H8C	109.5		

Symmetry codes: (i) *x*, −*y*+1/2, *z*−1/2; (ii) *x*, −*y*+1/2, *z*+1/2.
